# Unklare Ursache einseitiger Erblindung bei normwertigen Entzündungsparametern

**DOI:** 10.1007/s00393-024-01531-w

**Published:** 2024-06-14

**Authors:** Felix Müller, Christian Helmut Pfob, Matthias Wahle

**Affiliations:** 1 3. Med. Klinik, Sektion Rheumatologie & Klinische Immunologie, Universitätsklinikum Augsburg, Stenglinstr. 2, 86156 Augsburg, Deutschland; 2 Universitätsklinikum Augsburg, Nuklearmedizin, Medizinische Fakultät, Universität Augsburg, Stenglinstr. 2, 86156 Augsburg, Deutschland

**Keywords:** Riesenzellarteriitis, Arteriitis temporalis, Biomarker, Entzündungsparameter, Fallbericht, Giant cell arteritis, Temporal arteritis, Biomarkers, Inflammation parameters, Case report

## Abstract

Eine 70-jährige Patientin wird wegen akuter einseitiger Erblindung des rechten Auges vorgestellt. Bei unauffälligem C‑reaktivem Protein (CRP) und Blutsenkungsgeschwindigkeit (BSG) wird ein nichtarteriitischer embolischer Verschluss angenommen. Die weitere Anamnese ergibt jedoch den Verdacht auf eine Großgefäßvaskulitis, welcher durch die folgende bildgebende Diagnostik bestätigt wird. Dieser seltene Fall einer Großgefäßvaskulitis bei normwertigen Entzündungsparametern betont die Bedeutung von Anamnese und gezielter Diagnostik.

## Abstract (en)

A 70-year-old female patient presented with unilateral blindness of the right eye. As C‑reactive protein (CRP) and the erythrocyte sedimentation rate (ESR) were inconspicuous, a nonarteritic embolic occlusion was assumed; however, after detailed anamnesis large vessel vasculitis (LVV) appeared more likely, which was confirmed by the subsequent imaging diagnostics. This rare case of LVV without an increase in one of the inflammatory parameters CRP or ESR highlights the importance of the medical history and targeted diagnostic procedures.

## Anamnese

Eine 70-jährige Patientin wird wegen akut aufgetretener Erblindung des rechten Auges in der Notaufnahme vorgestellt und in der Klinik für Augenheilkunde aufgenommen. Der einseitigen Erblindung vorausgegangen waren Schmerzen von Schulter und Nacken, beginnend 2 Monate vor Aufnahme, zudem Kieferschmerzen bei längerem Kauen. Vier Wochen zuvor war eine plötzliche, passagere Diplopie aufgetreten. Die Akutdiagnostik im heimatnahen Krankenhaus ergab seinerzeit bei unauffälliger kranialer Computertomographie (cCT), unauffälliger nativer kranialer Magnetresonanztomographie (cMRT) und fehlenden laborchemischen Entzündungszeichen (s. Tab. 1) den Verdacht einer transitorisch ischämischen Attacke. Zwei Tage vor akuter Aufnahme bestand eine Amaurosis fugax des rechten Auges. Eine Allgemeinsymptomatik lag zu keiner Zeit vor.

An relevanten Nebenerkrankungen bestand eine arterielle Hypertonie. Kardiovaskuläre Ereignisse in der Vorgeschichte wurden nicht berichtet.

## Diagnostik

**Abb. 1:**
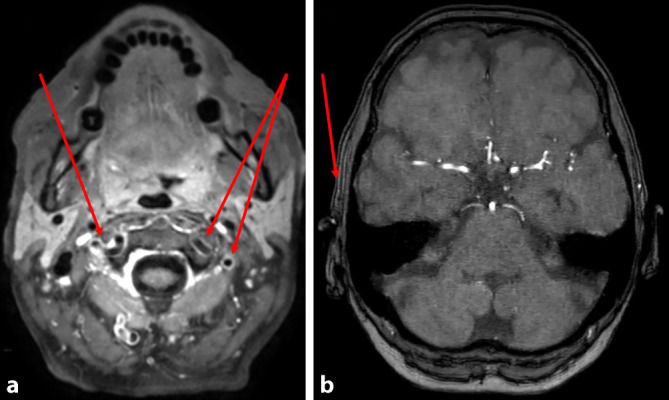
**a** cMRT, Sequenz T1 Black Blood transversal mit Kontrastmittel (KM). Deutliches KM-Enhancement der Vertebralarterien (*Pfeile*); **b** Sequenz 3D „time of flight“ (TOF), fehlendes Flusssignal der A. temporalis superficialis rechts (*Pfeil*)

**Abb. 2:**
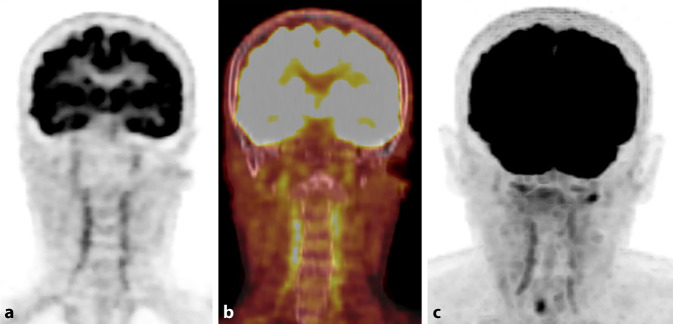
**a** [18F]-FDG-PET, koronar, „standardized uptake value“ (SUV) 7; **b** [18F]-FDG-PET/CT, koronar, Fusionsbild, SUV 5; **c** [18F]-FDG-PET, koronar, Maximumintensitätsprojektion (MIP), SUV 7. Deutliche Stoffwechselsteigerung der A. vertebralis beidseits

Bei Aufnahme beträgt die Sehschärfe rechts Handbewegungen, bei Entlassung nulla lux. Die Fundoskopie beschreibt eine ödematöse, blasse Papille, eine Tortuositas der Gefäße, eine Ischämiebande der inferioren Hälfte und einen Hemizentralarterienverschluss. CRP und BSG sind bei Aufnahme und im Verlauf normal (Tab. 1). CCT mit Angiographie an Tag 1 ohne Vaskulitis-typische Veränderungen. Die cMRT mit Kontrastmittel (KM) an Tag 3 weist eine Vaskulitis mit Manifestation in den Aa. temporales superficiales nach (Abb. 1). Die rechtsseitige A. temporalis superficialis sowie der Ramus frontalis der kontralateralen Arterie sind verschlossen. Die [18F]-FDG-PET/CT vom selben Tag ergibt Stoffwechselsteigerungen insbesondere in der A. vertebralis beidseits (Abb. 2) sowie geringer in der A. carotis interna, in der Bauchaorta und in den Beinarterien, gut vereinbar mit Riesenzellarteriitis. Eine Temporalisbiopsie wurde von der Patientin abgelehnt.

**Tab. 1:** Entzündungsparameter bei Diplopie 4 Wochen vor Erblindung (links) und bei Akutaufnahme wegen einseitiger Erblindung (rechts)

Vier Wochen zuvor (Diplopie)		Akute Erblindung		
Parameter	Tag −28	Tag 1	Tag 2	Tag 3
C‑reaktives Protein, mg/dl (NW < 0,5)	0,35	0,13	0,27	0,24
Blutsenkungsgeschwindigkeit (mm/h)	–	3	2	6
Leukozyten/nl (NW 3‑10/)	8,19	8,25	–	9,43

*NW* Normwert

## Diagnose

Großgefäßvaskulitis (Synonym: Riesenzellarteriitis [RZA]) mit Beteiligung supraaortaler Arterien und anteriorer ischämischer Optikusneuropathie (AION) bei normwertigen systemischen Entzündungsparametern (CRP/BSG).

## Therapie und Verlauf

Zunächst erfolgte eine Bolusgabe von Methylprednisolon 1000 mg i.v. an Tag 1 bis 3, gefolgt von Prednisolon (PDN) p.o. (Beginn mit 60 mg/Tag). Eine Verbesserung des Visus am rechten Auge trat nicht ein. Das linke Auge war weder zu Beginn noch im Verlauf betroffen. Eine steroidsparende Therapie (Methotrexat bzw. Tocilizumab) wurde empfohlen, von der Patientin jedoch abgelehnt. Die Reduktion von PDN wurde entsprechend den bekannten Behandlungsempfehlungen durchgeführt [4]. Weitere Manifestationen einer Großgefäßvaskulitis sind bisher nicht aufgetreten.

Differenzialdiagnostisch fanden sich keine Hinweise für eine infektiös bedingte Vaskulitis, eine andere entzündlich rheumatische Systemerkrankung, eine hereditäre Angiopathie oder eine IgG-4-assoziierte Erkrankung.

## Diskussion

Der vorliegende Fall beschreibt die zunächst uncharakteristisch und vieldeutig wirkende Manifestation einer seltenen entzündlichen Erkrankung. Dies hat Auswirkungen auf die Latenz von Beschwerdebeginn bis Diagnosestellung und damit wesentliche Konsequenzen auf Morbidität und Prognose. Es stellt sich die Frage, wie eine verzögerte Diagnosestellung bei Großgefäßvaskulitis in entsprechenden Konstellationen verhindert werden kann.

Die Großgefäßvaskulitis präsentiert sich zumeist mit suggestiver Leitsymptomatik und einer Erhöhung der systemischen Entzündungsparameter CRP und BSG. Diese sind in Verbindung mit der klinischen Symptomatik und der Bildgebung auch zur Fallklassifikation sowie der Definition von Remission und deren Verlaufskontrolle geeignet [4].

Isoliert normwertige CRP- oder BSG-Resultate werden in ca. 5–22 % der Fälle gemessen [5–9]. Dass sowohl CRP als auch BSG wie in diesem Fall normal gemessen werden, gilt als sehr selten (< 3 %).

Die sorgfältige Wertung der anamnestischen Angaben und eine nach Verfügbarkeit und Sensitivität bzw. Spezifität gestaffelte Diagnostik (klinische Untersuchung, farbkodierte Duplexsonographie [FKDS] der Kopf‑/Halsarterien und der A. axillaris, hochauflösende MRT der Aorta bzw. der Kopf‑/Halsarterien, [18F]-FDG-PET) erscheinen am ehesten geeignet, die Frühdiagnostik der Großgefäßvaskulitis zu verbessern [1, 2].

**Abb. 3:**
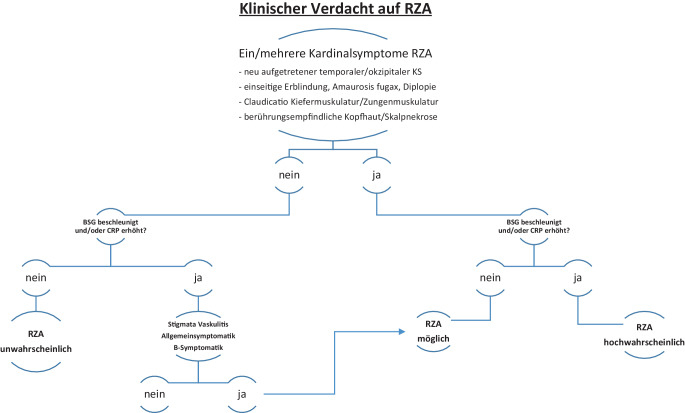
Flussschema zur Einschätzung der klinischen Wahrscheinlichkeit einer Riesenzellarteriitis

Wertvoll waren in diesem Fall eine rheumatologische Systemanamnese mit gezielter Abfrage der Leitsymptome einer Großgefäßvaskulitis sowie die strukturierte Bildgebung. Eine Strukturierung der Diagnostik durch Flussschemata hilft, die klinische Wahrscheinlichkeit einer Großgefäßvaskulitis einzuschätzen (s. Abb. 3).

Unklar ist noch, ob die laborchemische Erstdiagnostik und Verlaufskontrolle bei Großgefäßvaskulitis mit anderen Akute-Phase-Proteinen wie Komplementfaktoren oder Pentraxin‑3 [3, 10] optimiert werden können. Die Kontrolle der Bildgebung sollte unter Berücksichtigung der betroffenen Gefäßabschnitte in Frequenz und Methodenauswahl an die konkrete Situation angepasst werden. [18F]-FDG-PET-Untersuchungen zur Verlaufskontrolle sind in diesem Kontext nur in Subpopulationen der Betroffenen indiziert [11].

## Fazit für die Praxis

- Normwertige Entzündungsparameter schließen eine Großgefäßvaskulitis nicht aus und können daher zur deutlichen Diagnoseverzögerung führen.
- Eine gezielte Anamnese zu Leitsymptomen einer Großgefäßvaskulitis sollte bei Verdachtsmomenten erfolgen.
- Diagnosesicherung gelingt über objektivierbare Befunde wie FKDS, CTA, hochauflösende MRT/cMRT-Angiographie, [18F]-FDG-PET.
- Eine Strukturierung der Diagnostik durch Flussschemata hilft, die klinische Wahrscheinlichkeit einer Großgefäßvaskulitis einzuschätzen

## References

[1] Bley T, Guggenberger KV (2021) Kombinierte MRT/MRA für die Diagnostik der RZA. Z Rheumatol 80:559–562 34104995 10.1007/s00393-021-01021-3

[2] Christian D, Sofia R, Milena B et al (2023) EULAR recommendations for the use of imaging in large vessel vasculitis in clinical. practice, Bd. 2023. update. Annals of the Rheumatic Diseases:ard, S 2023–224543

[3] Conticini E, Hellmich B, Frediani B et al (2022) Utility of serum complement factors C3 and C4 as biomarkers during therapeutic management of giant cell arteritis. Scand J Rheumatol: 1–7 10.1080/03009742.2022.2047311 35383517

[4] Hellmich B, Agueda A, Monti S et al (2020) 2018 Update of the EULAR recommendations for the management of large vessel vasculitis. Ann Rheum Dis 79:19–30 31270110 10.1136/annrheumdis-2019-215672

[5] Laria A, Zoli A, Bocci M et al (2012) Systematic review of the literature and a case report informing biopsy-proven giant cell arteritis (GCA) with normal C‑reactive protein. Clin Rheumatol 31:1389–1393 22820967 10.1007/s10067-012-2031-3

[6] Martins P, Teixeira V, Teixeira FJ et al (2020) Giant cell arteritis with normal inflammatory markers: case report and review of the literature. Clin Rheumatol 39:3115–3125 32472460 10.1007/s10067-020-05116-1

[7] Naderi N, Mohammad AJ, Turesson C (2017) Large vessel involvement in biopsy-proven giant cell arteritis: incidence, distribution, and predictors. Scand J Rheumatol 46:215–221 27385090 10.1080/03009742.2016.1190984

[8] Parikh M, Miller NR, Lee AG et al (2006) Prevalence of a normal C‑reactive protein with an elevated erythrocyte sedimentation rate in biopsy-proven giant cell arteritis. Ophthalmology 113:1842–1845 16884778 10.1016/j.ophtha.2006.05.020

[9] Salvarani C, Hunder GG (2001) Giant cell arteritis with low erythrocyte sedimentation rate: frequency of occurrence in a population-based study. Arthritis Care & Research: Official Journal of the American College of Rheumatology 45:140–145 10.1002/1529-0131(200104)45:2<140::AID-ANR166>3.0.CO;2-2 11324777

[10] Tombetti E, Hysa E, Mason JC et al (2021) Blood Biomarkers for Monitoring and Prognosis of Large Vessel Vasculitides. Curr Rheumatol Rep 23:17 33569633 10.1007/s11926-021-00980-5 PMC7875948

[11] Van Der Geest KSM, Treglia G, Glaudemans A et al (2021) Diagnostic value of [18F]FDG-PET/CT for treatment monitoring in large vessel vasculitis: a systematic review and meta-analysis. Eur J Nucl Med Mol Imaging 48:3886–3902 33942141 10.1007/s00259-021-05362-8 PMC8484162

